# Characterization and Proteomic Analyses of the High Cr Resistance and Removability of a Novel *Lysinibacillus capsici* FPHNCRA4-48 Isolated from Highly Cr-Polluted Water

**DOI:** 10.3390/microorganisms14030611

**Published:** 2026-03-09

**Authors:** Dongmei Pan, Yinyan Chen, Zhijia Fang, Zhanghan Mo, Lukman Iddrisu, Mei Qiu, Qi Deng, Lijun Sun, Ravi Gooneratne

**Affiliations:** 1 College of Food Science and Technology, Guangdong Provincial Key Laboratory of Aquatic Product Processing and Safety, Guangdong Provincial Engineering Technology, Research Center of Marine Food, Key Laboratory of Advanced Processing of Aquatic Products of Guangdong Higher Education Institution, Guangdong Ocean University, Zhanjiang 524088, China; 15297720630@163.com (D.P.); rqxchenyinyan@163.com (Y.C.); 13318683281@163.com (Z.M.); lukmaniddrisu54@gmail.com (L.I.); qm08shj@163.com (M.Q.); jdoudengqi@163.com (Q.D.); suncamt@126.com (L.S.); 2 Department of Wine, Food and Molecular Biosciences, Lincoln University, Lincoln 7647, Canterbury, New Zealand; ravi.gooneratne@lincoln.ac.nz

**Keywords:** *Lysinibacillus capsici*, removal, Cr, extracellular polymeric substances (EPSs), proteomics

## Abstract

Chromium (Cr) is a common heavy-metal pollutant that poses a significant threat to both the environment and human health. Herein, a novel strain *Lysinibacillus capsici* FPHNCRA4-48, with a high Cr tolerance and removal performance, was isolated from Cr-contaminated plant water in Changde, Hunan Province. Structural characterization and proteomic analyses were performed to investigate the Cr removal performance and molecular mechanism of *L. capsici* FPHNCRA4-48. FPHNCRA4-48 can effectively remove more than 99% of the Cr(VI) at an initial concentration of 1000 μmol/L. The FTIR, 3D-EEM, and XPS results revealed that -OH, -NH_2_, and -CO-NH_2_ derived from extracellular polymeric substances (EPSs) were mainly involved in Cr(VI) removal. Interestingly, the protein content in the EPS increased significantly (1.32-fold) after exposure to Cr(VI). Moreover, proteomic analysis revealed that genes (rpmA, rpmI, rpmC, rplI, rpmD, deoB, deoC) related to translation and carbohydrate metabolism, and genes (pyk, icd, rpiB, eno) related to amino acid biosynthesis were all significantly up-regulated, suggesting that these pathways related to protein synthesis in *L. capsici* FPHNCRA4-48 were activated under Cr(VI) stress. Finally, KEGG ribosome pathway enrichment occurred. These data highlight the importance of microbial EPSs in bioremediation in Cr-polluted environments. This study identified highly efficient Cr(VI)-removing bacterial strains and conducted an in-depth analysis of the removal mechanism of their extracellular polymeric substances (EPSs), thereby providing theoretical foundations and technical support for the biological remediation of Cr(VI)-contaminated water bodies.

## 1. Introduction

Chromium (Cr) enters the ecosystem through various means, including the electroplating industry, stainless-steel production, leather tanning, and the production of dyestuffs and pigments, resulting in serious Cr pollution of water [1]. Cr occurs in various oxidation states, with hexavalent chromium (Cr(VI)) and trivalent chromium (Cr(III)) being the predominant forms found in the environment [2]. Cr(VI) is both mutagenic and carcinogenic [3]. Even at very low doses, Cr(VI) may pose significant threats to the human body, leading to lung cancer, nasopharyngeal cancer, liver and kidney damage, and skin ulcers [4]. Therefore, the effective control of Cr pollution has become a pressing issue. Bioremediation is an eco-friendly alternative method for removing heavy-metal pollutants [5]. Microorganisms act as adsorbents and play a major role in environmental pollution control. The adsorption of heavy metals by microorganisms occurs mainly through complexation, precipitation, and electrostatic attraction [6]. The studies on the removal of Pb, Cr, and Cd by *Megasporum*, *Aspergillus niger*, and *Penicillium* were noted [7,8]. There have been some advances in research on microorganisms for Cr tolerance and Cr removal. Das et al. [9] explored the process by which *Bacillus amyloliquefaciens*, a newly identified bacterium with a high tolerance for chromium, reduces and eliminates Cr(VI) from environments contaminated with chromite soils. Extracellular polymeric substances (EPSs) are macromolecular organic substances secreted outside the cell body by bacteria under certain environmental conditions, as the main component of resistance to metal toxicity for many microorganisms. Studies have shown that both microorganisms and their EPSs have strong adsorption properties for heavy metals [10]. However, in most cases, the molecular mechanism of Cr(VI) removed by microbial EPSs requires further exploration.

In this study, an excellent strain *L. capsici* FPHNCRA4-48 with Cr(VI)-chelating ability was isolated from Cr-contaminated water samples using the BPR-Cr(VI) plate overlay method. Furthermore, the Cr(VI) removal ability of the isolate and its EPS was explored using multispectral techniques. Finally, proteome sequencing was performed to analyze the mechanism of the EPS increase in response to Cr(VI) using DIA (Data-Independent Acquisition Proteomics) technology. This study aimed to provide a theoretical basis and technical support for the control of Cr(VI) pollutants using Cr(VI) removal strains.

## 2. Materials and Methods

### 2.1. Chemicals and Reagents

Luria Bertani (LB) broth and agar were purchased from Beijing Landbridge Technology Co., Ltd. (Beijing China). Potassium dichromate (K_2_Cr_2_O_7_) was purchased from the Shantou Guanghua Chemical Factory (Shantou, China). Disodium ethylenediaminetetraacetic acid (EDTA-2Na) and cetyltrimethylammonium bromide (CTAB) were purchased from Sinopharm Chemical Reagent Co., Ltd. (Shanghai, China). Bromophthalate Red (BPR) was purchased from Shanghai Yiji Industry Co., Ltd. (Shanghai China).

### 2.2. Isolation and Identification of Cr(VI)-Chelating Strains

The BPR-Cr(VI) detection solution was prepared according to a previous study [11]. Potassium chromate solution, 0.1% CTAB solution, sodium acetate to acetic acid buffer (pH = 5.5), and 0.05% BPR solution were mixed in a 1:3:3:2 ratio, and each solution was added sequentially and shaken thoroughly. Water samples were collected from a mining area in Changde City, Hunan Province (29.81° N, 111.55° E). A 100 mmol/L K_2_Cr_2_O_7_ solution was used as the Cr(VI) stock solution. This stock solution was added in varying amounts to LB liquid medium to create gradient media with Cr(VI) concentrations ranging from 50 μmol/L to 1500 μmol/L. This gradient was employed for the initial screening of Cr(VI)-tolerant strains. The strains were isolated, purified, and glycerol-preserved for further experiments. The isolates were stained with the above test solution after growth in solid medium, followed by determination of the diameter of the chelation circle around the colony. The isolate with the largest chelation circle was amplified by PCR using the 16S rRNA universal primers 27F: (5′-AGAGTTTGATCCTGGCTCAG-3′) and 1492R: (5′-GGTTACCTTGTTACGACTT-3′), and the results were sent to Sangon Biotech (Shanghai) Co., Ltd. (Shanghai, China). A phylogenetic tree was then created using MEGA software (Molecular Evolutionary Genetics Analysis, v.6.0).

### 2.3. Cr(VI) Sensitivity Analysis of the Isolates

Bacterial sensitivity represents a method of inhibiting the growth of certain bacteria in response to adverse conditions, such as the presence of heavy metals or alterations in growth parameters like changes in medium composition [12]. Imaging grayscale heatmaps of bacterial colony growth at different Cr(VI) concentrations visually demonstrate variations in resistance gradients. The sensitivity of the isolates to Cr(VI) was assessed using a spot t assay [13]. The isolates were cultured in LB medium at 37 °C for 18-24 h to 2 × 10^9^ cells/mL (optical density of 1 at 600 nm), and the bacterial solutions were diluted with sterile water in a 10-fold gradient to 10^−1^, 10^−2^, 10^−3^, and 10^−4^. Next, 5 μL of the diluted bacterial solutions was spotted on LB agar with gradually increasing Cr(VI) concentrations (0, 500, 1000, 1500, 2000, and 2500 μmol/L) and then incubated for 1–2 days in a gel imaging system to observe the growth phase. In addition, the strains were inoculated with 2% inoculum in LB broth containing the same concentration gradients of Cr(VI), and the optical density (OD) was measured at 600 nm every 12 h to evaluate their sensitivity to Cr(VI) [14].

### 2.4. Microscopic Analyses for L. capsici FPHNCRA4-48

In order to assess alterations in cellular appearance following Cr(VI) uptake, the bacteria were nurtured within LB medium containing Cr(VI) at concentrations of 0 and 2000 µmol/L for a duration of 2 days. Cell precipitates were collected by centrifugation at 4500× *g* for 10 min at 4 °C, and they were washed gently with phosphate buffer solution (PBS) to remove the culture medium. The remaining solid samples were mixed with 2.5% glutaraldehyde solution and fixed at 4 °C for 12 h [15]. Following the fixation, the precipitates were centrifuged and washed three times with PBS to remove the glutaraldehyde solution. The cells were then dehydrated sequentially using 30%, 50%, 70%, 80%, 90%, 95%, and 100% ethanol solutions in a gradient, and finally freeze-dried for observation using a scanning electron microscope (SEM) (Carl Zeiss Microscopy GmbH, Oberkochen, Germany). The elemental compositions on the surface were examined through Energy-Dispersive X-ray Spectroscopy (EDS). SEM was used to observe the cell morphology after Cr(VI) stress, while EDS was employed to verify the presence of Cr elements on the cell surface.

### 2.5. Removal Performance of Cr(VI) by L. capsici FPHNCRA4-48

The recovered *L. capsici* FPHNCRA4-48 was inoculated with 2% inoculum volume and incubated at 37 °C and 135 r/min with the specified Cr(VI) levels (0, 500, 1000, 1500, 2000, and 2500 μmol/L). No metal addition was used as a control group to determine the effect of the medium components on the removal of Cr(VI). The OD_600_ was measured at 0, 12, 24, 36, 48, 60, and 72 h. Three replicates were performed for each group. After centrifugation (12,000 r/min, 10 min), the Cr(VI) concentration in the supernatant was determined by dibenzoyl dihydrazide spectrophotometry. It was presumed that the adsorption onto the cells accounted for the discrepancy between the initial and residual concentrations of the metal ions [16]. Cr(VI) removal efficiency was calculated according to the following equation (Equation (1)):

(1) $$R\;=\;(1\;-\;\frac{C_{2}}{C_{1}})\;\times \;100\%$$

where R is the removal rate of Cr(VI), %; *C*_1_ is the initial concentration of Cr(VI), μmol/L; and *C*_2_ is the supernatant concentration of Cr(VI) in the medium, μmol/L.

### 2.6. EPS Extraction and Compositional Analysis

*L. capsici* FPHNCRA4-48 cells were incubated aerobically in LB broth at 3% inoculum for 3 days within Cr(VI) concentrations (0, 500, 1000, 1500, 2000, and 2500 μmol/L). The supernatant was separated by centrifugation at 5000 r/min for a duration of 10 min at a temperature of 4 °C. Subsequently, an equivalent volume of ethanol, pre-chilled to −20 °C, was mixed with the supernatant and incubated at −20 °C for a period of 24 h to facilitate the precipitation of EPS. The precipitates were collected by centrifugation of the alcohol-sedimented mixture at 9000 r/min for 30 min at 4 °C, and were dissolved in distilled water to obtain crude EPS. Finally, the solution was loaded into a dialysis bag (7000 Da), placed in ultrapure water, and dialyzed at 4 °C for 48 h. Finally, the solution in the dialysis bag was lyophilized to obtain pure EPS [17].

The polysaccharide, protein, and DNA contents of the extracted EPS were determined. The polysaccharide content was determined using the phenol sulfate method [18]. The protein concentration was assessed utilizing the Bradford assay [19]. Meanwhile, the DNA concentration was evaluated through the colorimetric diphenylamine test [20].

### 2.7. Adsorption Kinetics and Isothermal Adsorption Experiments

We added 400 mg/L of the raw EPS to the flasks in a volume of 50 mL (pH = 6.0 ± 0.1), with a final Cr(VI) concentration of 2000 μmol/L. The flasks were shaken at 30 °C and 120 r/min for 10, 30, 60, 90, 120, 150, 180, 240, 300, 360, and 400 min. Subsequently, the solution was dialyzed for 12 h in a large beaker containing 500 mL of deionized water using a 7000 Da dialysis bag. Samples were collected, and the supernatant Cr(VI) concentration was determined using dibenzoyl dihydrazide spectrophotometry.

We combined 10 mL of K_2_Cr_2_O_7_ solution and 0.5 mg EPS in a 50 mL conical flask with various Cr(VI) concentrations of 125, 250, 500, 750, 1500, 2000, and 2500 μmol/L (pH = 6.0 ± 0.1), and those were cultivated in a shaker at 30 °C, 120 r/min for 1 h. Afterward, the solution was introduced into a dialysis tube, which was subsequently immersed in a beaker filled with 200 mL of water for a period of 12 h. The external liquid in the dialysis bag was collected to determine the Cr(VI) concentration.

The adsorption kinetics of Cr(VI) on EPS were evaluated using the pseudo-first-order kinetic model (Equation (2)), pseudo-second-order kinetic model (Equation (3)), and Elovich kinetic model (Equation (4)), as follows:

(2) $$q_{t}\;=\;q_{e}(1\;-\;e^{-K_{1}t})$$

(3) qt=k2 qet21+k2 qet

(4) $$q_{t}=\frac{1}{\beta }\;\ln\left(\alpha \beta \right)+\frac{1}{\beta }\ln t$$

where q_t_—adsorption capacity of EPS for Cr(VI) at time t (mg/g); q_e_—adsorption capacity of EPS at adsorption equilibrium (mg/g); K_1_—rate constant of the proposed first-order kinetic model (min^−1^); K_2_—rate constant of the proposed second-order kinetic model (g/(mg·min)^−1^); α—initial adsorption rate (mg·g^−1^·min^−1^); β—desorption constant (g·mg^−1^); t—adsorption time (min).

To further evaluate the removal performance of EPS for Cr(VI), adsorption isotherms were used. The Langmuir isotherm model and the Freundlich isotherm model are commonly used to analyze the interaction between the adsorbent and adsorbate. The Langmuir isotherm model depicts an ideal adsorption model of a monomolecular layer where adsorption occurs on a uniform surface [21]. The Freundlich isotherm model is an empirical model that is mainly used to describe the non-ideal adsorption processes occurring on heterogeneous surfaces with various types of active sites.

Langmuir isothermal adsorption model:

(5) $$q_{e}=\frac{q_{m}\;K_{L}\;C_{e}}{1+K_{L}\;C_{e}}$$

(6) $$\frac{C_{e}}{q_{e}}=\frac{C_{e}}{q_{m}}+\frac{1}{q_{m}\;K_{L}}$$

where q_e_—adsorption of Cr(VI) by EPS when adsorption reaches equilibrium (mg/g); q_m_—maximum adsorption capacity when adsorption occurs (mg/g); C_e_—concentration of Cr(VI) in solution when adsorption reaches equilibrium (mg/L); K_L_—adsorption equilibrium constant (L/mg).

Freundlich isothermal adsorption model:

(7) $$q_{e}=K_{F}\;{C_{e}}^{1/n}$$

(8) $$\ln q_{e}=\frac{1}{n}\;\ln C_{e}+\ln\;K_{F}$$

where q_e_—adsorption of Cr(VI) by EPS when adsorption reaches equilibrium (mg/g); C_e_—concentration of Cr(VI) when adsorption reaches equilibrium (mg/L); K_F_—Freundlich’s constant (mg·g^−1^) (L·mg^−1^)^1/n^; n—coefficient of relationship between adsorption capacity and adsorption rate, and the influence factor of inhomogeneity in the reaction system.

### 2.8. EPS Characterization Techniques

#### 2.8.1. Three-Dimensional Fluorescence Spectroscopy (3D-EEM) Analysis

Three-dimensional EEM is an effective instrumental method for identifying chromophores and is widely used in chemical and biochemical studies of molecular structure and function [22]. The fluorescence absorption characteristics of *L. capsici* FPHNCRA4-48 EPS in the presence or absence of Cr(VI) were measured using EEM (F-7000) with an excitation wavelength scanning range of 200–500 nm and an emission wavelength scanning range of 250–600 nm. The interval of excitation wavelength was set at 10 nm, and the interval of the emission wavelength was set at 5 nm. The widths of the excitation and emission slits were fixed at 5 nm.

#### 2.8.2. Fluorescence Spectral Analysis

Fluorescence spectrometry is a spectral analysis technique for qualitative and quantitative analysis based on the property of emission of fluorescence by molecules of a substance after absorption of light energy. The fluorescence spectrometer (RF-5301PC) from Kyoto, Japan (Shimadzu Corporation) was utilized to set the excitation wavelength at 280 nm and the range of emission wavelength at 300–450 nm for the fluorescence detection of the lyophilized EPS at the concentrations of Cr(VI) (0, 500, 1000, 1500, and 2000 μmol/L).

#### 2.8.3. Fourier Transform Infrared Spectroscopy (FTIR) Analysis

Infrared spectroscopy is a commonly used method for organic functional group identification and structural analysis. The Fourier Transform Infrared Spectroscopy (FTIR) model TENSOR27 was utilized to determine the alterations in the principal functional groups of the extracellular polymeric substances (EPSs), both in the presence and absence of Cr(VI) treatment. The EPS was combined with potassium bromide (KBr) at a mass ratio of 1 to 150. The FTIR scanned spectral range was 400 cm^−1^–4000 cm^−1^ with a resolution of 4 cm^−1^ and 32 scans.

#### 2.8.4. X-Ray Photoelectron Spectroscopy (XPS) Analysis

XPS is a technique used to identify the chemical state and elemental composition of a material’s surface, which allows for the identification and quantification of elements and functional groups on a sample’s surface by analyzing the binding energy of the photoelectrons. Using a Thermo Scientific K-Alpha XPS instrument (Waltham, MA, USA) with Al Kα rays as the excitation source, the photoelectron spectra of the core energy levels of C 1s, N 1s, and O 1s of EPS under 0 and 1500 μmol/L Cr(VI) stress were analyzed to identify the functional groups on the surface of the samples [23].

### 2.9. Proteome Sequencing Analysis of L. capsici FPHNCRA4-48 EPS

Proteomics is an effective research tool to analyze the biological processes and cellular composition of microorganisms under heavy metal stress, and to reveal the intrinsic mechanisms by studying changes in gene or protein expression. The EPSs from *L. capsici* FPHNCRA4-48 under 0 and 1500 μmol/L Cr(VI) stress were extracted, corresponding to the A0 (A1, A2, A3) and B1500 (B1, B2, B3) groups, respectively. Each treatment was performed in triplicate. Proteins were isolated by the addition of 400 μL RIPA lysis buffer, and the concentration of proteins in the samples was measured through the Bicinchoninic Acid method. Proteins that were expressed differentially were identified by utilizing the chi-squared statistical test, with a *p*-value < 0.05 and multiplicity of difference greater than 4 as up-regulated expression and less than 0.25 as down-regulated expression [24]. The effects of Cr(VI) on the proteins from FPHNCRA4-48 EPS were investigated using the DIA proteomics technique of bioinformatics, including protein functional annotation, functional enrichment analysis, and functional feature-based clustering analysis.

### 2.10. Statistical Analysis

One-way ANOVA was used to analyze the significance of differences between groups using GraphPad Prism software. *p* < 0.05 indicates statistically significant differences, indicated by “*”. GraphPad Prism 8.0.2 and Origin 2021 software were used for plotting.

## 3. Results and Discussion

### 3.1. Isolation, Identification, and the Tolerance of the Isolate

**Figure 1:**
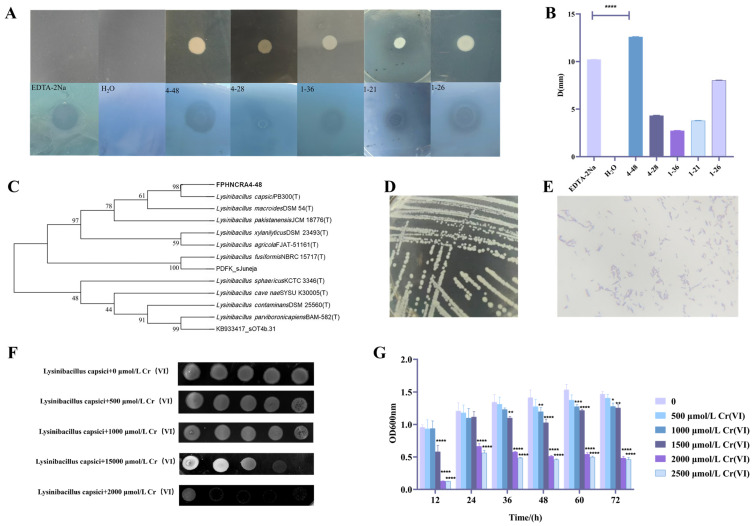
Isolation and characterization of *L. capsici* FPHNCRA4-48. (**A**) Screening of strains for chelating circles by the BPR-Cr(VI) plate overlay method; (**B**) chelation circle diameter of the strain (total diameter minus colony diameter), **** *p* < 0.0001; (**C**) phylogenetic tree of strain FPHNCRA4-48; (**D**) morphological characteristics of colonies; (**E**) Gram staining microscopy results of the strain; (**F**) growth of 10-fold serial dilutions of *L. capsici* FPHNCRA4-48 on LB solid medium with 0–2000 μmol/L Cr(VI); (**G**) growth of *L. capsici* FPHNCRA4-48 on LB liquid medium with 0–2000 μmol/L Cr(VI). Comparison with the control group (0 mmol/L Cr(VI) group), * *p* < 0.05, ** *p* < 0.01, *** *p* < 0.001, **** *p* < 0.0001.

One hundred and sixty Cr(VI)-tolerant strains were isolated from polluted water samples from a mining site in Changde City, and strain 4-48 appeared to have the largest chelating circle of 12.6 mm via the BPR-Cr(VI) plate overlay method, suggesting the strongest Cr(VI) chelation capacity (Figure 1A,B). According to 16S rRNA sequencing and homology comparison, strain 4-48 had 99.31% homology with *Lysinibacillus capsici* (PB300). A phylogenetic tree was constructed using MEGA 5.05 software (Figure 1C). The strain was identified as *Lysinibacillus capsici* and named FPHNCRA4-48. Gram staining revealed a purple color, indicating it is a Gram-positive bacterium. The cells were arranged in straight rods and chains using a 100x oil microscope (Figure 1D,E).

The sensitivity of *L. capsici* FPHNCRA4-48 to Cr(VI) was assessed by observing the growth in LB solid and liquid media at Cr(VI) concentrations of 0-2000 μmol/L. *L. capsici* grew optimally in the absence of Cr(VI), while the OD values decreased gradually with the increase in Cr(VI) concentration. The growth of *L. capsici* was significantly inhibited in solid medium when the Cr(VI) concentrations were 2000 and 2500 μmol/L, and the cells were slow or even unable to grow when the OD values were less than 0.5 in the liquid medium (Figure 1F,G). The Cr(VI)-resistant strain *P. fluorscens* had bacterial growth that was directly inhibited when the Cr(VI) concentration in the growth medium reached 3 mmol/L [25], whereas *L. capsici* FPHNCRA4-48 exhibited a half-maximal inhibitory concentration at 1.5 mmol/L of Cr(VI), with growth inhibition observed at 2.5 mmol/L. These results indicate that *L. capsici* FPHNCRA4-48 is resistant to Cr(VI) and may play a role in the removal of Cr(VI).

### 3.2. Removal of Cr(VI) by L. capsici FPHNCRA4-48

**Figure 2:**
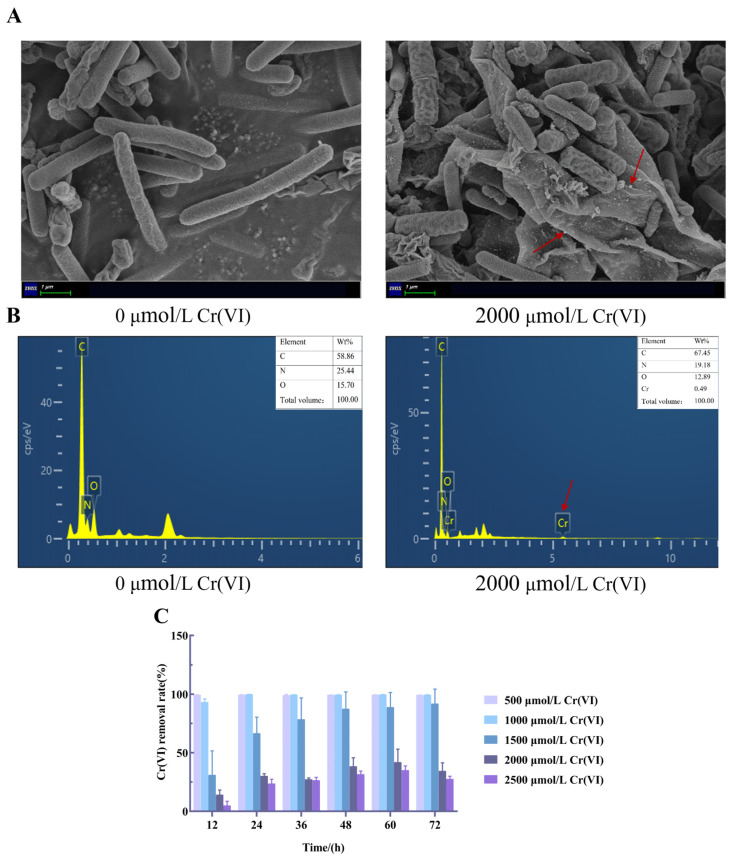
Distribution of Cr(VI) and Cr(VI) removal capacity of *L. capsici* FPHNCRA4-48. (**A**) SEM plots of 0 μmol/L and 2000 μmol/L Cr(VI)-treated *L. capsici* FPHNCRA4-48; (**B**) EDS plots of 0 μmol/L and 2000 μmol/L Cr(VI)-treated *L. capsici* FPHNCRA4-48; (**C**) Cr(VI) removal of *L. capsici* FPHNCRA4-48 at different Cr(VI) concentrations.

In the absence of Cr(VI), the cells appeared as short rods with a length of approximately 5 μm using SEM, with a smooth surface and regular shape. As shown in Figure 2A, after exposure to 2000 μmol/L Cr(VI), the cells appeared to have a few irregular depressions, obvious folds, and an increase in extracellular secretion of mucilage, which was similar to *Rhodopseudomonas acidophila* treated with Cr [26]. Some insoluble chromium particles were visible on the *L. capsici* FPHNCRA4-48 surface (shown by red arrows). In addition, the mass percentages of C, N, O, and Cr were 67.45%, 19.18%, 12.89%, and 0.49%, respectively (Figure 2B). EDS analysis of Cr(VI)-exposed *L. capsici* FPHNCRA4-48 revealed characteristic Cr peaks, and the presence of Cr indicated adsorption of Cr(VI) or Cr(III) products on the bacterial cell surface [27]. Therefore, we hypothesized that bacterial extracellular secretion plays an important role in the removal of Cr(VI).

We set out to investigate the removal efficiency of Cr(VI) by *L. capsici* FPHNCRA4-48 at various concentrations of Cr(VI) (0, 500, 1000, 1500, 2000, 2500 μmol/L). *L. capsici* FPHNCRA4-48 exhibited a good removal ability of Cr(VI), with a 99% removal rate at the initial concentrations of Cr(VI) of 500 μmol/L and 1000 μmol/L (Figure 2C). However, when the initial concentration of Cr(VI) increased to 2000 and 2500 μmol/L, the removal rate significantly decreased to 34.38% and 27.58%, respectively. Among all tested concentrations, the highest removal rate (99%) was achieved at an initial Cr(VI) concentration of 1000 μmol/L. The Cr minimum inhibitory concentration of *Azotobacter* s8 exceeded 100 mg/L CrCl_3_, with a Cr removal rate of 10.53% at an initial concentration of 50 mg/L [28]. In contrast, the *L. capsici* FPHNCRA4-48 studied here achieved a removal rate exceeding 99% at the same Cr(VI) concentration. The results indicated that the *L. capsici* FPHNCRA4-48 strain can effectively remove Cr(VI) from aqueous solutions.

Microbial cell walls are selectively permeable, and they regulate the entry and exit of various substances. The toxicity of high-concentration metals to microorganisms leads to the denaturation of intracellular proteins and inactivation of bioenzymes, and it inhibits the reproduction and metabolism of microorganisms [29]. When the Cr(VI) concentration reached 34.76 mg/L, the self-protection mechanisms of *Geloina erosa* were damaged or even collapsed, rendering them unable to carry out normal metabolic activities and reproduction [30]. High Cr(VI) concentrations (>50 mg/L) compromise cell membrane integrity, whereas EPS protects cells by preferentially adsorbing Cr(VI), thereby reducing intracellular Cr(VI) accumulation. Consequently, *L. capsici* FPHNCRA4-48 exhibits strong resistance and a high Cr(VI) removal efficiency, likely due to the removal effect of extracellular substances such as bacterial EPSs.

### 3.3. Adsorption Kinetics and Isothermal Adsorption

The Cr(VI) adsorption behavior onto *L. capsici* FPHNCRA4-48 EPS was analyzed using fitting kinetic models, such as the pseudo-first-order kinetic model and the pseudo-second-order kinetic model. The correlation coefficient (R^2^) of the proposed primary kinetic equation for the adsorption of Cr(VI) by EPS was 0.814, which was lower than the proposed secondary correlation coefficient of 0.855. Therefore, the proposed secondary kinetic equation better reflects the process of Cr(VI) adsorption by EPS. These results indicate that the adsorption of Cr(VI) by EPS is mainly attributed to chemical action.

The Elovich model exhibited a significantly higher correlation coefficient (R^2^ = 0.912) for fitting the kinetic data of EPS adsorption of Cr(VI) compared to the pseudo-first-order (R^2^ = 0.814) and pseudo-second-order kinetic models (R^2^ = 0.855). This indicates a highly heterogeneous distribution of surface energy on EPS, with the adsorption process jointly governed by chemical reactions and diffusion factors. This aligns with the complex biochemical composition of EPS (multicomponent structure including proteins and polysaccharides), indicating that the adsorption mechanism is not solely chemisorption but rather a heterogeneous diffusion process involving multi-site coordination. Furthermore, the α value is significantly greater than the β value (Table S1), suggesting that the adsorption rate far exceeds the desorption rate, demonstrating the strong irreversibility of the EPS adsorption process for Cr(VI).

**Figure 3:**
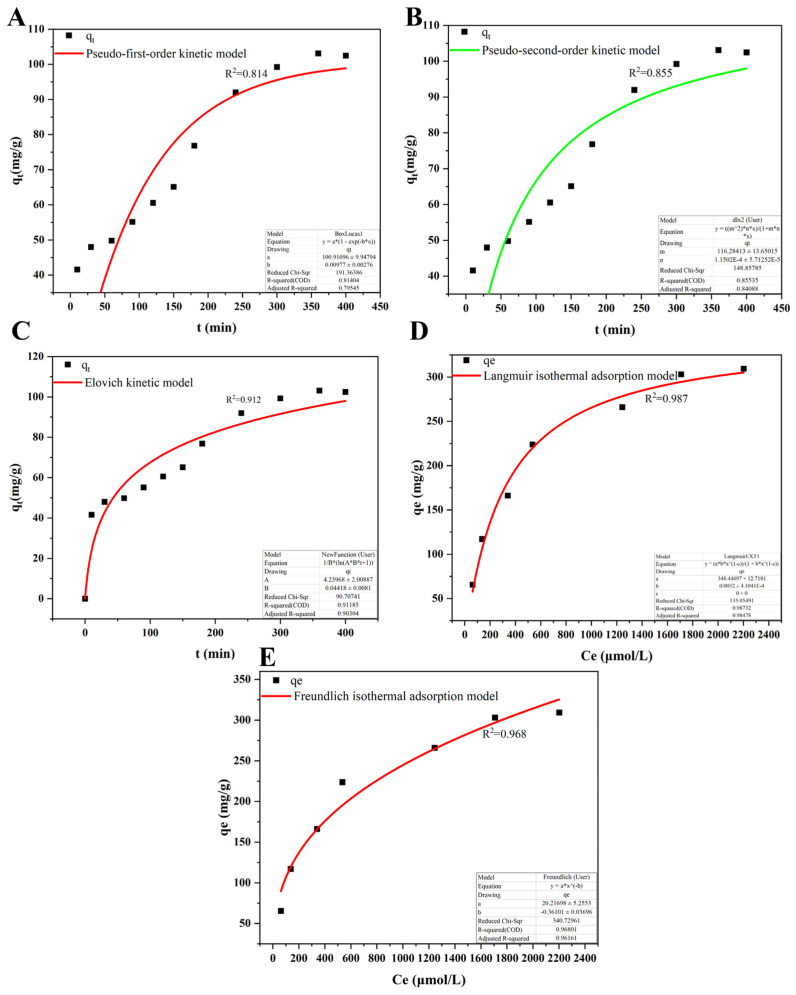
EPS kinetics and isothermal adsorption diagram. (**A**) Pseudo-first-order kinetic model of adsorption of Cr(VI) by EPS; (**B**) pseudo-second-order kinetic model for the adsorption of Cr(VI) by EPS; (**C**) Elovich kinetic model for the adsorption of Cr(VI) by EPS; (**D**) Langmuir isothermal adsorption model for Cr(VI) adsorption by EPS; (**E**) Freundlich isothermal adsorption model for Cr(VI) adsorption by EPS.

In the present study, the adsorption mechanism of *L. capsici* FPHNCRA4-48 EPS on Cr(VI) was investigated using Langmuir and Freundlich adsorption isotherms (Table S2). While EPS was adsorbing Cr(VI), the correlation coefficients for both the Langmuir and the Freundlich models, when applied to the data, were higher than 0.9, which indicated that the adsorption process conformed to both models. The R^2^ coefficients of the Langmuir and the Freundlich adsorption isotherm models were 0.987 and 0.968 (Figure 3D,E), respectively. Overall, the Langmuir isothermal adsorption model more accurately describes the process of Cr(VI) adsorption by EPS.

### 3.4. Characterization of Cr(VI) Removal by L. capsici FPHNCRA4-48 EPS

#### 3.4.1. Three-Dimensional EEM Analysis and Fluorescence Spectroscopy of EPS Following Cr(VI) Exposure

**Figure 4:**
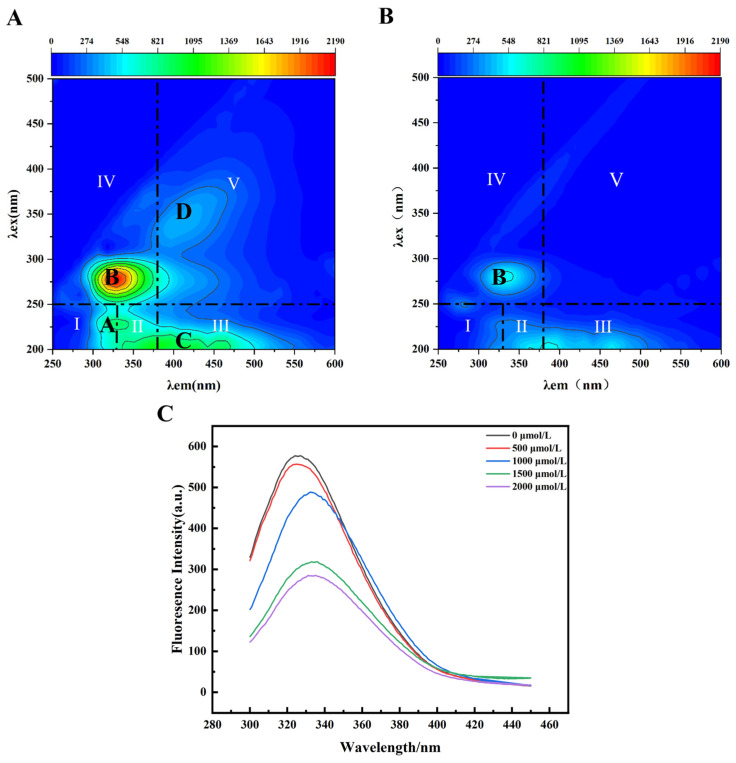
Three-dimensional EEM, fluorescence spectroscopy, and FTIR analysis of *L. capsici* FPHNCRA4-48 EPS after exposure to Cr(VI). (**A**) EEM profiles of EPS extracted from the strain cultured under an initial Cr(VI) concentration of 0 μmol/L; (**B**) EEM profiles of EPS extracted from the strain cultured under an initial Cr(VI) concentration of 1500 μmol/L; (**C**) fluorescence spectra of EPS extracted from strains cultured at initial Cr(VI) concentrations of 0, 500, 1000, 1500, and 2000 μmol/L.

Three-dimensional EEM was used to analyze the EPS fluorescent substances in the presence and absence of Cr(VI). The positions of fluorescence peaks for various substances are as follows: Class I: Casein, excitation wavelength (Ex) range: 200–250 nm, emission wavelength (Em) range: 300–330 nm; Class II: Tryptophan, Ex range: 200–250 nm, Em range: 330–380 nm; Class III fulvic acid substances: Ex range 200–250 nm, Em range 380–500 nm; Class IV: Soluble microbial substances, Ex range 250–400 nm, emission wavelength range 300–380 nm; Class V: Humic substances, Ex range 250–400 nm, Em range 380–500 nm. The fluorescent compounds in the pristine EPS of *L. capsici* FPHNCRA4-48 showed that Ex/Em at 285/350 nm (peak A) and 278/327 nm (peak B) corresponded to protein-like substances such as tyrosine and tryptophan, respectively [31]. In addition, two significantly weaker peaks were detected at the Ex/Em wavelengths of 210/400 nm (Peak C) and 343/411 nm (Peak D), characterizing fulvic acid-like substances and humic acid-like substances, respectively (Figure 4A). According to Figure 4B, tryptophan, tyrosine, fulvic acid-like substances, and soluble microbial-like substances all showed weakening fluorescence intensities in the EPS after 1500 μmol/L Cr(VI) treatment, and the weakening from tryptophan-like proteins was the most clear. Zhang et al. [32] found that the light intensities of peaks A and B in biofilm EPSs were burst by Cu(II), indicating that tryptophan-like and aromatic proteins were associated with Cu(II) with a strong binding capacity. Therefore, tryptophan-like and aromatic-like proteins may complex with Cr(VI) during the bioabsorption of Cr(VI). Moreover, after exposure to Cr(VI), the intensity of peak D in the EPS weakened, suggesting that a burst between Cr(VI) and humic substances occurred through electrostatic interactions.

Tryptophan, which is a part of EPS, has often been noted to be instrumental in the reduction of Cr(VI) as indicated by 3D-EEM assessments [33]. Tryptophan-like protein substances are capable of being transformed into aromatic ring compounds through oxidation by strong oxidizing agents, creating a protective barrier that serves as a defense mechanism for microorganisms against adverse environmental conditions. Tryptophan-like substances have also been reported to account for a certain percentage of dissolved organic matter that can bind heavy metals [34]. Therefore, the 3D-EEM results showed that the protein component of EPS, as the main Cr(VI)-resistant reactive substance, was complexed with Cr(VI).

To further explore the interaction between EPS and Cr(VI), fluorescence spectroscopy was performed. With the increase in Cr(VI) concentration (0–2000 μmol/L), the fluorescence intensity of EPS gradually weakened and a red-shift phenomenon appeared, which indicated that Cr(VI) interacted with the fluorescent material, resulting in a fluorescence burst (Figure 4C). These findings are in agreement with the 3D-EEM results, indicating an interaction with Cr(VI).

#### 3.4.2. The Primary Functional Groups Involved in the Cr(VI) Adsorption

**Figure 5:**
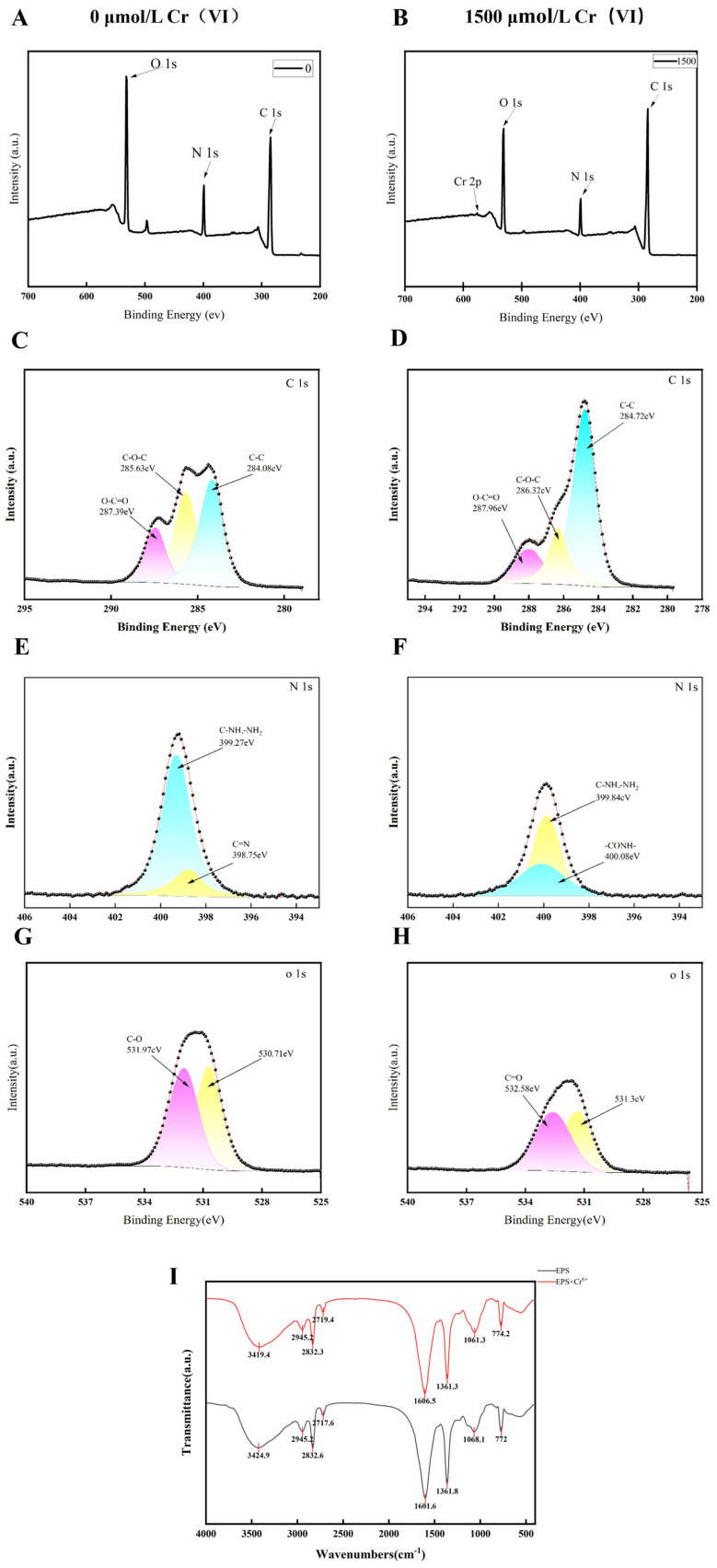
XPS spectroscopic analysis of extracted *L. capsici* FPHNCRA4-48 EPS in the presence and absence of Cr(VI) conditions. (**A**) Full-band XPS spectra of EPSs extracted under Cr(VI)-free conditions; (**B**) XPS full-band spectra of EPS extracted at an initial concentration of 1500 μmol/L for Cr(VI); (**C**,**E**,**G**) elemental scanning analysis of C1s, N1s, and O1s of EPS extracted at an initial concentration of Cr(VI) of 0 μmol/L is shown, respectively; (**D**,**F**,**H**) elemental scanning analysis of C1s, N1s, and O1s of EPS extracted at an initial concentration of Cr(VI) of 1500 μmol/L is shown, respectively; (**I**) FTIR of EPS at Cr(VI) concentrations of 0 and 1500 μmol/L.

The elemental contents of the EPS were obtained by XPS analysis. The XPS full-band spectra are shown in Figure 5A,B, suggesting the presence of Cr(VI) after exposure to Cr(VI). Considering that each peak within the XPS spectrum is indicative of an electron with a distinctive binding energy associated with a specific element, detailed high-resolution scans and deconvolution analyses were conducted for the C 1s, O 1s, and N 1s regions to glean comprehensive insights into the chemical functionalities present within the EPS.

As shown in Figure 5, after Cr(VI) treatment, the C-C content increased from 40.62% to 64.04%, and the C-O-C and O-C=O contents decreased from 38.78% and 20.6% to 21.01% and 14.95%, respectively. This indicated that sugar glycosides and hemiacetal groups generated hydroxyl groups (RCOH) during the adsorption of Cr(VI). The functional groups of carboxyl groups and hemiacetals in EPS play key roles in the reduction of heavy metals [35]. The content of amino nitrogen in the N 1s spectral region of EPS increased after Cr(VI) treatment, and the C=N structure may have undergone hydrolysis or oxidation and converted to an amide structure (-CONH-). Cr(VI) reduction may be achieved via participation of amine groups within microbial EPS. The nitrogen atom in the amine group possesses a lone pair of electrons, exhibiting strong reducing properties that can reduce Cr(VI) to trivalent chromium. The amine group itself is oxidized to products such as imine (-C=N-) or amide (-CO-NH-), which are then presumed to form insoluble chromium hydroxide precipitates like such as Cr(OH)_3_ (Figure 5E,F). Jeff discovered that Pseudomonas bacteria (strain CRB5) isolated from an abandoned wood preservative treatment plant could tolerate Cr(VI) concentrations as high as 520 mg/L and reduce the toxic Cr(VI) into insoluble Cr(III) precipitates [36]. The O 1s peak of EPS can be decomposed into two peaks (Figure 5G,H). Treatment with Cr(VI) led to a shift in the peak of C=O to a higher binding energy region by 0.59 eV, which corresponded to an increase in the binding energy of the peak of C-O-C/C-O-H by 0.61 eV. The increase in the specific C-O-H/C-O-C moiety but the decrease in C=O could be attributed to the fact that polysaccharides are also involved in Cr(VI) reduction. The high-resolution XPS spectrum of Cr 2p is shown in Figure S1. A Cr(III) 2p_1_/_2_ peak appears at 577 eV, and a Cr(VI) 2p_3_/_2_ peak appears at 587 eV. This indicates that part of the Cr(VI) is adsorbed as Cr(VI), while another part is reduced to Cr(III), with reduction being the primary process.

The conformational changes in the functional groups of *L. capsici* FPHNCRA4-48 EPS under 0 and 1500 μmol/L Cr(VI) stress were investigated by FTIR analysis (Figure 5I). The absorption peaks in the Cr(VI)-treated EPS in the range of 400–4000 cm^−1^ were significantly changed compared with the control group. The peak at 3424.9 cm^−1^ after Cr(VI) adsorption was shifted to 3419.4 cm^−1^, which is attributed to the N-H stretching in proteins. This absorption peak is related to the N-H stretching vibration in proteins and the O-H stretching vibration in sugars [37]. It is deduced that the interactions between O-H and N-H groups with Cr(VI) are part of the Cr(VI) adsorption process, and that -OH and -NH_2_ present on the cell surface might have a direct interaction with Cr(VI). Additionally, hydroxyl and carboxyl groups are implicated in the reduction of Cr(VI) [38]. The absorption peak of EPS at 1601.6 cm^−1^ was significantly shifted after Cr(VI) adsorption, which indicated the presence of -NH bending vibration and C-N stretching vibration in the amide II band from the protein [39].

Combined with the XPS and FTIR results, functional groups such as -OH, -NH_2_, and -CO-NH_2_ derived from *L. capsici* FPHNCRA4-48 EPS play an important role in the removal of Cr(VI), which are related to the mechanism of Cr(VI) reduction and adsorption.

#### 3.4.3. Analysis of EPS Compositions

**Figure 6:**
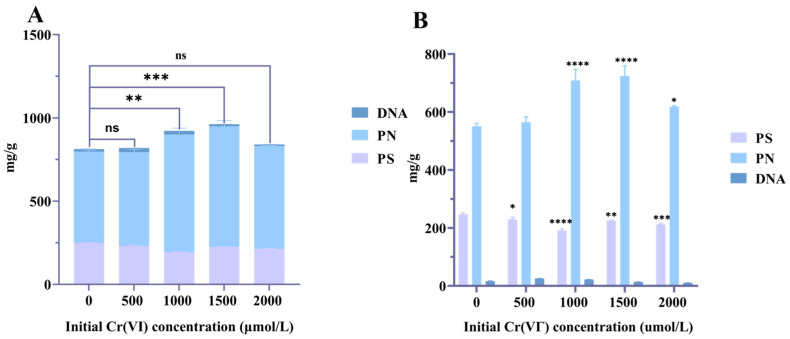
Analysis of EPSs Content and Composition at Different Initial Cr(VI) Concentrations. * *p* < 0.05, ** *p* < 0.01, *** *p* < 0.001, **** *p* < 0.0001, ns indicates no significant difference *p* > 0.05. (**A**) EPSs content at different initial Cr(VI) concentrations; (**B**) PS, PN, and DNA contents in EPSs with different initial Cr(VI) concentrations.

The contents of EPS obtained were 811.71 mg/g, 817.94 mg/g, 920.71 mg/g, and 961.0 mg/g when the initial concentrations of Cr(VI) were 0, 500, 1000, and 1500 μmol/L, respectively (Figure 6A), which showed that the EPS content increased with an increasing Cr(VI) concentration. Bacteria tend to produce more EPS for survival after being exposed to seawater media containing toxic metals and chemicals [40]. Additionally, the elevated protein content in EPS may stem from high concentrations of Cr(VI) disrupting cell membrane integrity, leading to non-specific leakage of intracellular proteins [41].

The increase in the protein (PN) component produced by *L. capsici* FPHNCRA4-48 under Cr(VI) stress was much larger than that in the polysaccharide (PS) component (Figure 6B). As the Cr(VI) concentration increased from 0 μmol/L to 1500 μmol/L, the PN content increased from 549.47 mg/g to 723.72 mg/g (1.32-fold). There was little change in the nucleic acid (DNA) content.

The changes in response to Cr(VI) from *L. capsici* FPHNCRA4-48 manifested as an increase in total EPS and protein fractions. After exposure to Cr(VI), *L. capsici* secreted more PN and PS to resist Cr(VI) toxicity because EPS contained some exoenzymes that could reduce heavy metal ions [42,43]. Increased PN production is thought to be a protective strategy against Cr(VI) stress in bacteria [44]. Thus, the proteins from *L. capsici* EPS, being the main components of coping with Cr(VI) stress, have an important role in Cr(VI) removal.

## 4. Proteomic Analysis of *L. capsici* FPHNCRA4-48 EPS

### 4.1. Differential Protein Analysis

Proteomic studies were conducted to investigate the adaptive response mechanism of Cr(VI) adsorption by *L. capsici* EPS. Up to 1073 proteins were identified by DIA proteomics technology analysis, of which 1072 were quantified. The sample correlation and PCA analysis (Figure S2A,B) showed a high correlation between the two samples and a relatively high similarity of the samples within the group. The replication of protein identification in each subgroup was observed using a Venn diagram (Figure S2C), which showed that there were 962 shared proteins between the two groups, and 12 and 98 proteins were specifically expressed in groups A0 and B1500, respectively. The total number of differentially expressed proteins was 269, with 170 up-expressed and 99 down-expressed (Figure S2D). The differentially expressed proteins were shown in the form of volcano plots (Figure S2E), indicating changes in the expression of multiple proteins in *L. capsici* FPHNCRA4-48 under Cr(VI) stress.

### 4.2. GO Analysis

**Figure 7:**
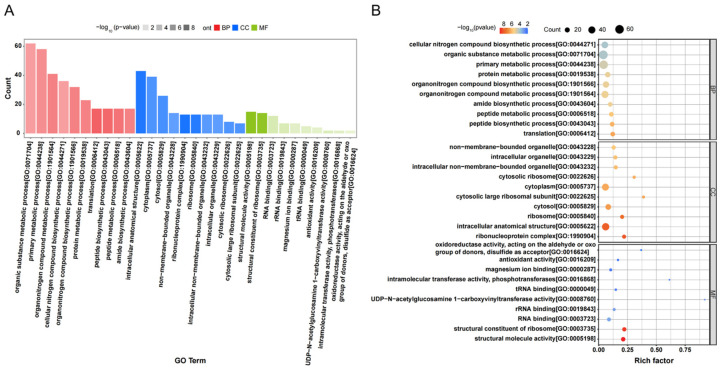
GO analysis of *L. capsici* FPHNCRA4-48 EPS. (**A**) Classification histogram for GO analysis of differentially expressed proteins (A0 vs. B1500); (**B**) classification bubble diagram for GO enrichment analysis of differentially expressed proteins.

The Gene Ontology (GO) is comprised of three principal components: Molecular Function (MF), Cellular Component (CC), and Biological Process (BP) [45]. There were a total of 1613 annotated genes (1453 up-regulated and 160 down-regulated). The histogram of the GO enrichment analysis (Figure 7A) showed that the differentially expressed proteins were mainly involved in the organic metabolic process and primary metabolic process of BP, the intracellular anatomical structure and cytoplasm of CC, and the structural molecular activity and structural components of the ribosome of MF. From the *p*-value of the differential expression gene function enrichment analysis, it can be inferred that the greatest variability occurred in the GO terms of biological process CC, which indicated that CC was the category with the greatest variation in biological function. Of these, the most affected are ribonucleoprotein complexes, followed by intracellular anatomical structures. In CC, the number of differentially expressed proteins in intracellular anatomical structures and the cytoplasm was relatively high, with 43 and 39, respectively (Table S3), which indicated a critical role in the change in biological functions.

The results of the top 10 significantly enriched GO items by *p*-value are shown in Figure 7B. The differentially expressed proteins were mainly enriched in the protein translation process, peptide biosynthesis process, ribonucleoprotein complexes, intracellular anatomical structures, structural molecular activities, and structural components of ribosomes. Of these, the structural molecular activities and ribosomal structural components differed significantly. Moreover, proteins related to peptide biosynthesis processes were significantly up-regulated (17 up-regulated versus 0 down-regulated). These data demonstrate that the peptide synthesis of EPS in *L. capsici* FPHNCRA4-48 was boosted in response to Cr(VI) stress.

### 4.3. KEGG Analysis

**Figure 8:**
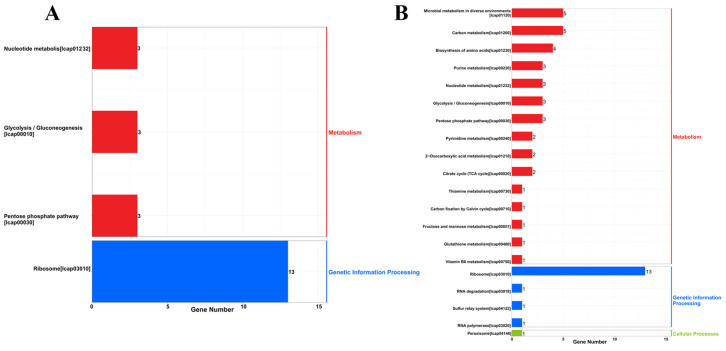
KEGG analysis of *L. capsici* FPHNCRA4-48 EPS. (**A**) Categorical histogram of KEGG metabolic pathways of differentially expressed proteins; (**B**) analysis of the top 20 KEGG enrichment pathways for differential proteins.

The KEGG analysis of *L. capsici* EPS showed that genes related to the ribosomal and pentose phosphate pathways were significantly enriched, especially in the ribosomal pathway, of which the rich factor value (degree of enrichment) was close to 0.22 (Figure S3). Presumably, these pathways played an important role in the Cr(VI) removal process. Proteomics technology showed that protein expression changes were found in *Serratia marcescens* CM01 under Cr(VI) stress, and the pentose phosphate pathways in the KEGG were significantly enriched with related up-regulated genes [46]. *L. capsici* was less enriched for glycolytic and nucleotide metabolic pathways. The pathways of differentially expressed proteins were mainly enriched in metabolic pathways (13), ribosomes (13), biosynthesis of secondary metabolites (6), carbon metabolism (5), biosynthesis of amino acids (4), and biosynthesis of cofactors (4). As shown in the Figure 8A, the differentially expressed proteins in the KEGG were mainly annotated in four pathways: nucleotide metabolism (3), glycolysis (3), pentose phosphate pathway (3), and ribosome (13).

Thirteen differential genes of the ribosomal pathways were enriched in the genetic information processing of KEGG, which is significantly higher than the other pathways [47]. Gang et al. [48] mentioned that significantly enriched ribosomes were important pathways in KEGG involved in structural molecular activities of proteins contributing to the structural integrity of the complexes, especially ribosomes. Ribosomes are the source of protein synthesis in cells and play an important role in protein synthesis in organisms [49]. This implies that protein synthesis processes were significantly activated in *L. capsici* FPHNCRA4-48. Additionally, ribosomes synthesized a plethora of proteins to preserve cellular integrity and functionality, mend impaired proteins, and generate novel proteins to counteract against Cr(VI)-induced stress. This was consistent with the increase in proteins produced by *L. capsici* under Cr(VI) stress. In the ribosomal pathway, genes related to ribosomal proteins such as rpmC and rplO are significantly up-regulated in *L. capsici*. The gene rpmC encoding a ribosomal protein is part of the 50S ribosomal subunit and plays a key role in ribosome assembly and function, especially in maintaining ribosome structure and stability during translation. RpmC is involved in the synthesis and regulation of ribosomal proteins in conjunction with other genes (rplO and rplF) [50]. Enhanced ribosomal function and accelerated protein synthesis related to Cr(VI) adsorption or reduction are the central molecular mechanisms for *L. capsici* to cope with Cr(VI) stress.

The pathways related to energy metabolism in *L. capsici* were also affected by Cr(VI) stress. In the pentose phosphate pathway, the deoB gene showed an up-regulation trend. KEGG enrichment results showed that the differentially expressed proteins were significantly enriched in metabolic pathways and ribosomes, suggesting that material synthesis and carbon metabolism were altered in *L. capsici* under Cr(VI) stress. This confirmed that *L. capsici* facilitated the secretion of proteins and polysaccharides under Cr(VI) stress.

Genes related to translation and carbohydrate metabolism, such as deoB, deoC, rpmD, rplU, and rpmA, were significantly differentially expressed in the top 20 KEGG pathways of the differential proteins (Figure 8B). Genes related to amino acid biosynthesis pyk, icd, rpiB, and eno were up-regulated and expressed, with rpiB being significantly up-regulated (*p* < 0.01), indicating that protein synthesis and expression were up-regulated in *L. capsici* under Cr(VI) stress. In addition, glutathione (GSH) metabolism genes, such as icd in *L. capsici*, were up-regulated following Cr(VI) exposure. Kilic et al. [51] applied a proteomic approach to study the proteotoxic stress response of Cr(VI) on *Pseudomonas aeruginosa*. The up-regulation of proteins in the glutathione system, outer membrane proteins involved in the detoxification of free radicals, suggested a resistant mechanism to Cr. Tripathi et al. [52] found that the high level of GSH in *Coccidioides* after arsenic exposure inhibited cellular oxidative stress. GSH can protect yeast cells from heavy metal toxicity by chelating them directly to form GSH-metal complexes [53]. Therefore, differential expression of genes related to translation, carbohydrate metabolism, and amino acid biosynthesis, together with synergistic activation of glutathione metabolic pathways, constitutes a complex adaptive mechanism of enhanced protein synthesis and oxidative stress defense in response to Cr (VI) in *L. capsici*.

### 4.4. Protein Interaction Analysis

**Figure 9:**
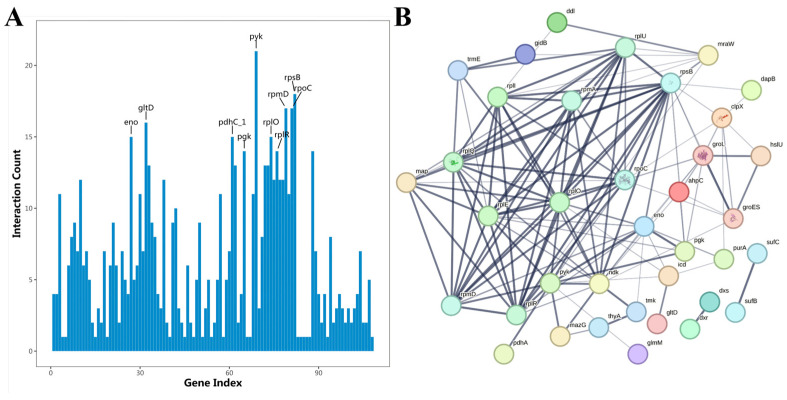
PPI analysis of *L. capsici* FPHNCRA4-48 EPS. (**A**) Histogram of interaction connectivity of group A0 to B1500 proteins; (**B**) PPI interaction network diagram.

Protein–protein interaction (PPI) network analysis is an important aspect of proteomics. The STRING database (v11, string-db.org) was used as the data source for constructing PPI networks [54]. A schematic diagram of the network interactions between these differentially expressed proteins was constructed (Figure 9B).

The up-regulated proteins rpmA and rpmD with high FC values (8.01 and 8.07, respectively) coordinate the functions of multiple proteins through an extensive interaction network involved in key pathways (e.g., ribosomal function, metabolic regulation) related to Cr(VI) reduction. The high clustering in the interaction networks suggested that rpmA and rpmD played an important role in protein translation, stress response, and metabolic regulation. Moreover, protein catabolism and synthesis can also repair some damaged proteins or generate new proteins to resist Cr(VI) stress. The up-regulated proteins deoxyribose-5-phosphate aldolase deoB, nucleoside diphosphate kinase ndk, and adenine deaminase ade were mainly enriched in the purine metabolism pathways. Differential proteins involved in purine metabolism may also be related to the mechanism of bacterial response to Cr(VI) stress [55]. Four genes in the amino acid biosynthesis pathway are up-regulated, including icd ribulose-5-phosphate isomerase B, which provides energy to the cell (NADH) and can act as an electron donor for the reduction of Cr(VI) [56].

As Figure 9A shows, there are up to 21 proteins that interact with pyk (eno, pgk, groEL, glmM, rplQ, rplO, rpmD, rplR, rplE, mazG, yjbM, ahpC, pdhA, pdhC_1, pdhD, rpsB, ndk, yedJ, bfmBAB, bfmBC, deoC). Pyruvate kinase, encoded by pyk, is a key rate-limiting enzyme in the glycolysis pathway. The up-regulation of pyk increases the amount and activity of the enzyme, catalyzing the conversion of more phosphoenolpyruvate (PEP) into pyruvate and significantly accelerating the rate of glycolysis. Upon pyk up-regulation, glycolysis is accelerated to produce more pyruvate, which can further enter the tricarboxylic acid cycle (TCA cycle) and thoroughly oxidize and decompose to produce a large amount of ATP for the cells [57]. Likewise, *L. capsici* needs to consume a large amount of energy to cope with Cr(VI) stress, including actively transporting Cr(VI) out of the cell and synthesizing detoxification-related substances. Intermediates produced during glycolysis, as well as pyruvate, can be involved in EPS synthesis as carbon sources and precursors [58]. For example, pyruvic acid can be converted into sugars, amino acids, and other substances for EPS synthesis, thereby enhancing Cr(VI) removal through the formation of EPS complexes.

The rpmA protein is a ribosomal protein (Ribosomal Protein L27) involved in the assembly of ribosomes and protein translation processes. Once ribosome function is affected by Cr(VI) stress, *L. capsici* can adapt to the extreme environment by maintaining the protein translation stability of rpmA. Thus, rpmA can enhance the removal capacity of EPS by enhancing the polysaccharides and proteins of EPS, which contribute to the adsorption of Cr(VI). The rpmD protein is another ribosomal protein (Ribosomal Protein L30) involved in the structure and function of ribosomes, maintaining ribosome stability, and ensuring efficient protein translation. By maintaining ribosomal function, rpmD may promote the production of proteins related to EPS synthesis, thereby enhancing the secretion or adsorption properties of EPS. Duffitt et al. [59] indicated that the expression of rpmC was synchronously upregulated with DNA repair and membrane protein genes in *Escherichia coli* under soil stress, forming a synergistic response network.

## 5. EPS-Mediated Cr(VI) Removal Mechanisms

**Figure 10:**
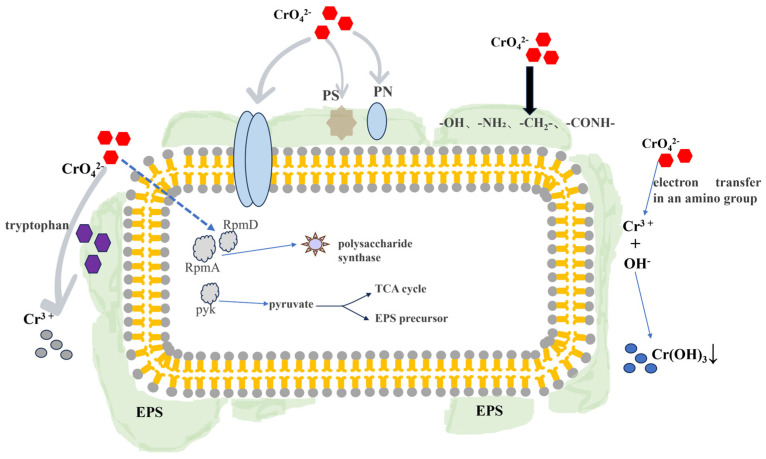
Diagram of the mechanism of Cr(VI) removal by *L. capsici* FPHNCRA4-48.

This study investigated the mechanism of Cr(VI) removal from the perspective of extracellular polymeric substances (EPSs) and employed dynamic ion analysis (DIA) technology for proteomic sequencing analysis to elucidate the molecular mechanism of EPS adsorption. The strain adsorbs Cr(VI) through three pathways: First, proteins, polysaccharides, and key functional groups (such as -OH, -NH_2_, and -CO-NH_2_) within the EPS secreted by the strain participate in Cr(VI) removal, while outer membrane proteins engage in radical detoxification, restricting Cr(VI) entry into cells. Second, tryptophan mediates the reduction of Cr(VI) to Cr(III). Third, an amine electron transfer mechanism occurs: -NH_2_ becomes protonated and adsorbs the Cr(VI) anion (CrO_4_^2−^), subsequently acting as an electron donor to reduce Cr(VI) to Cr(III) (Figure 10).

*L. capsici* FPHNCRA4-48 mediates Cr(VI) removal through its microbial EPS, with a core mechanism dominated by reduction and supplemented by adsorption. Key interaction sites include -OH, hemiacetal, glycosidic, and carboxyl groups derived from polysaccharides in EPSs, along with -NH_2_, N-H, and -CONH- functional groups from protein sources: On the one hand, the lone pair electrons on the nitrogen atom of amino groups confer strong reducing properties, predominantly reducing Cr(VI) to Cr(III) while oxidizing themselves to imine or amide structures. -OH and -COOH synergistically participate in the reduction reaction. On the other hand, -OH and -NH_2_ adsorb and immobilize Cr(VI) through hydrogen bonding, electrostatic interactions, and surface complexation. The reduced Cr(III) ultimately forms precipitates. This mechanism is further confirmed by the detection of characteristic Cr(III) peaks via XPS, the shift of functional group characteristic peaks observed in FTIR, and the structural changes in functional groups characterized by XPS.

## 6. Conclusions

By integrating Cr(VI) tolerance with EPS-mediated Cr(VI) removal mechanisms, this study successfully screened chromium-tolerant strains from chromium-contaminated water samples. Subsequently, strains exhibiting Cr(VI) chelation capacity were identified as *L. capsici* FPHNCRA4-48 through the BPR-Cr(VI) plate overlay method. This screening method demonstrates considerable innovation. In vitro environmental tests revealed that *L. capsici* FPHNCRA4-48 exhibits a potent Cr(VI) removal capacity. This study investigated the mechanism by which the strain removes Cr(VI) from the perspective of extracellular polymeric substances (EPSs). Dynamic ion analysis (DIA) technology was employed for proteomic sequencing analysis, revealing the molecular mechanism by which EPS removes Cr(VI).

Under Cr(VI) stress, EPS secretion significantly increased, with the most pronounced increase observed in protein content. Proteomics analysis revealed that protein translation, peptide biosynthesis, ribonucleoprotein complex assembly, and protein adsorption functions within the EPS of FPHNCRA4-48 were significantly upregulated following Cr(VI) exposure. This upregulation primarily occurred through nucleotide metabolism (ndk, ade, dut), glycolysis (pyk, eno, pdhA), the pentose phosphate pathway (deoB, rpiB, deoC), translation and carbohydrate metabolism (deoB, deoC, rplU), amino acid biosynthesis (pyk, icd, rpiB), and ribosome (rpmA, rpmC, rplO, rpmD) pathways. This indicates that under Cr(VI) stress, *L. capsici* FPHNCRA4-48 enhances protein synthesis and expression by upregulating translation-related genes to counteract Cr(VI) toxicity. The findings confirm that the EPSs secreted by this strain adsorb Cr(VI) and participate in its reduction to Cr(III) precipitates, playing a crucial role in mitigating Cr(VI) toxicity. This establishes a foundation for utilizing an EPS as a biosorbent to achieve the bioremediation of Cr(VI)-contaminated water bodies.

## Data Availability

The original data presented in the study are openly available in ProteomeXchange Consortium at PXD075244 [60,61].

## Supplementary Materials

The following supporting information can be downloaded at https://www.mdpi.com/article/10.3390/microorganisms14030611/s1, Table S1: Kinetic modeling parameters for Cr(VI) adsorption by EPS; Table S2: Parameters of Langmuir and Freundlich models for Cr(VI) adsorption by EPS; Table S3: Table of results of functional enrichment analysis of differentially expressed genes; Figure S1: XPS spectroscopic analysis of extracted *L. capsici* FPHNCRA4-48 EPS in the presence of Cr(VI) conditions. The elemental scanning analysis of Cr 2p of EPS extracted at an initial concentration of Cr(VI) of 1500 μmol/L; Figure S2: Basic Proteomic Sample Characteristics; (A) Heat map of inter-sample correlation; (B) Scatterplot of PCA scores for all samples; (C) Venn Diagram for Repeatability Analysis of Intergroup Sample Identification; (D) Histogram of the distribution of the number of differentially expressed proteins in different comparison groups; (E) Group A0 vs. Group B1500 Volcano Map; Figure S3: Enrichment analysis of KEGG metabolic pathway for differentially expressed proteins: bubble map.
