# Supplementary material for: Characterization and Proteomic Analyses of the High Cr Resistance and Removability of a Novel Lysinibacillus capsici FPHNCRA4-48 Isolated from Highly Cr-Polluted Water
Source: Microorganisms. 2026 Mar 9;14(3):611. doi: 10.3390/microorganisms14030611 (PMC13028638; doi:10.3390/microorganisms14030611)
Supplement: Supplementary file 1 [file microorganisms-14-00611-s001.zip › microorganisms-4178504-supplementary.pdf]

Supplementary Materials for

Characterization and proteomic analyses on the high Cr resistance and removability of a novel *Lysinibacillus Capsici* FPHNCRA4-48 isolated from highly Cr-polluted water

Dongmei Pan [1], Yinyan Chen [1], Zhijia Fang [1, *], Zhanghan Mo [1], Lukman Iddrisu [1], Mei Qiu [1], Qi Deng [1], Lijun Sun [1], and Ravi Gooneratne [2]

1 College of Food Science and Technology, Guangdong Provincial Key Laboratory of Aquatic Product Processing and Safety, Guangdong Provincial Engineering Technology, Research Center of Marine Food, Key Laboratory of Advanced Processing of Aquatic Products of Guangdong Higher Education Institution, Guangdong Ocean University, Zhanjiang, 524088, China; 15297720630@163.com (D.P.); rqxchenyinyan@163.com (Y.C.); 13318683281@163.com(Z.M.); lukmaniddrisu54@gmail.com(L.I.) ; qm08shj@163.com (M.Q.); jdoudengqi@163.com(Q.D.) ; suncamt@126.com(L.S.)

2 Department of Wine, Food and Molecular Biosciences, Lincoln University, Lincoln, Canterbury 7647, New Zealand; ravi.gooneratne@lincoln.ac.nz

* Correspondence: fangzj@gdou.edu.cn; Tel.: +86-0759-2396027

Table S1. Kinetic modeling parameters for Cr(VI) adsorption by EPS

Pseudo-first-order kinetic model			Pseudo-second-order kinetic model			Elovich kinetic model		
q_{max}(mg/g)	K_1(min^{-1})	R^2	K_2(g/mg/min)	q_{max}(mg/g)	R^2	β	α	R^2
119.895	0.0117	0.814	0.0001	120.482	0.855	0.054	2.425	0.912

Table S2. Parameters of Langmuir and Freundlich models for Cr(VI) adsorption by EPS

metal ion	Langmuir isothermal adsorption model			Freundlich isothermal adsorption model		
	q_{max}(mg/g)	K_L(L/mg)	R^2	$K_F((mg·g^{-1})(L·mg^{-1})^{1/n})$	1/n	R^2
Cr(Ⅵ)	351	0.062	0.987	47	0.421	0.968

Table S3. Table of results of functional enrichment analysis of differentially expressed genes （The top ten in each category with the smallest P-value）

GO.ID	Term	Count	Ontology	P-Value
GO:0006412	translation	17	BP	0.00000034
GO:0043043	peptide biosynthetic process	17	BP	0.00000064
GO:0006518	peptide metabolic process	17	BP	0.0000016
GO:0043604	amide biosynthetic process	17	BP	0.0000062
GO:1901564	organonitrogen compound metabolic process	41	BP	0.0000062
GO:1901566	organonitrogen compound biosynthetic process	32	BP	0.0000074
GO:0019538	protein metabolic process	23	BP	0.0000076
GO:0044238	primary metabolic process	58	BP	0.000021
GO:0071704	organic substance metabolic process	62	BP	0.000074
GO:0044271	cellular nitrogen compound biosynthetic process	36	BP	0.000082
GO:1990904	ribonucleoprotein complex	13	CC	0.0000000065
GO:0005622	intracellular anatomical structure	43	CC	0.00000001
GO:0005840	ribosome	13	CC	0.000000019
GO:0005829	cytosol	26	CC	0.00000023
GO:0022625	cytosolic large ribosomal subunit	7	CC	0.0000004
GO:0005737	cytoplasm	39	CC	0.00000042
GO:0022626	cytosolic ribosome	8	CC	0.00000045
GO:0043232	intracellular non-membrane-bounded organelle	13	CC	0.000001
GO:0043229	intracellular organelle	13	CC	0.0000013
GO:0043228	non-membrane-bounded organelle	14	CC	0.0000018
GO:0005198	structural molecule activity	15	MF	0.00000000091
GO:0003735	structural constituent of ribosome	14	MF	0.0000000019
GO:0003723	RNA binding	12	MF	0.00058
GO:0019843	rRNA binding	7	MF	0.00081
GO:0008760	UDP-N-acetylglucosamine 1-carboxyvinyltransferase activity	2	MF	0.00107
GO:0000049	tRNA binding	5	MF	0.00298
GO:0016868	intramolecular transferase activity, phosphotransferases	2	MF	0.00315
GO:0000287	magnesium ion binding	7	MF	0.00371
GO:0016209	antioxidant activity	4	MF	0.00509

continuation sheet

GO.ID	Term	Count	Ontology	P-Value
GO:0016624	oxidoreductase activity, acting on the aldehyde or oxo group of donors, disulfide as acceptor	2	MF	0.01005

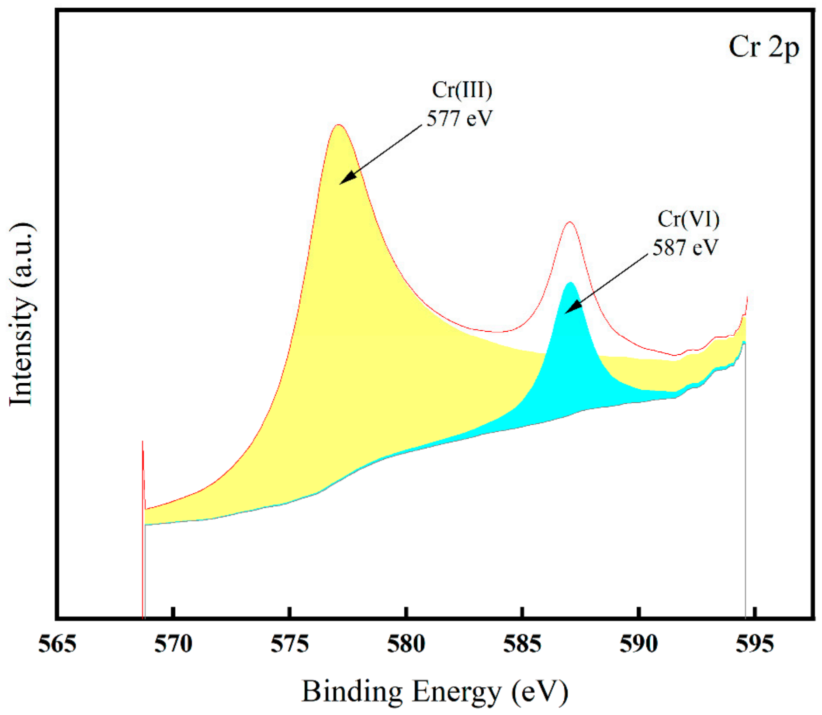

Figure S1. XPS spectroscopic analysis of extracted *L. capsici* FPHNCRA4-48 EPS in the presence of Cr(VI) conditions. The elemental scanning analysis of Cr 2p of EPS extracted at an initial concentration of Cr(VI) of 1500 μmol/L.

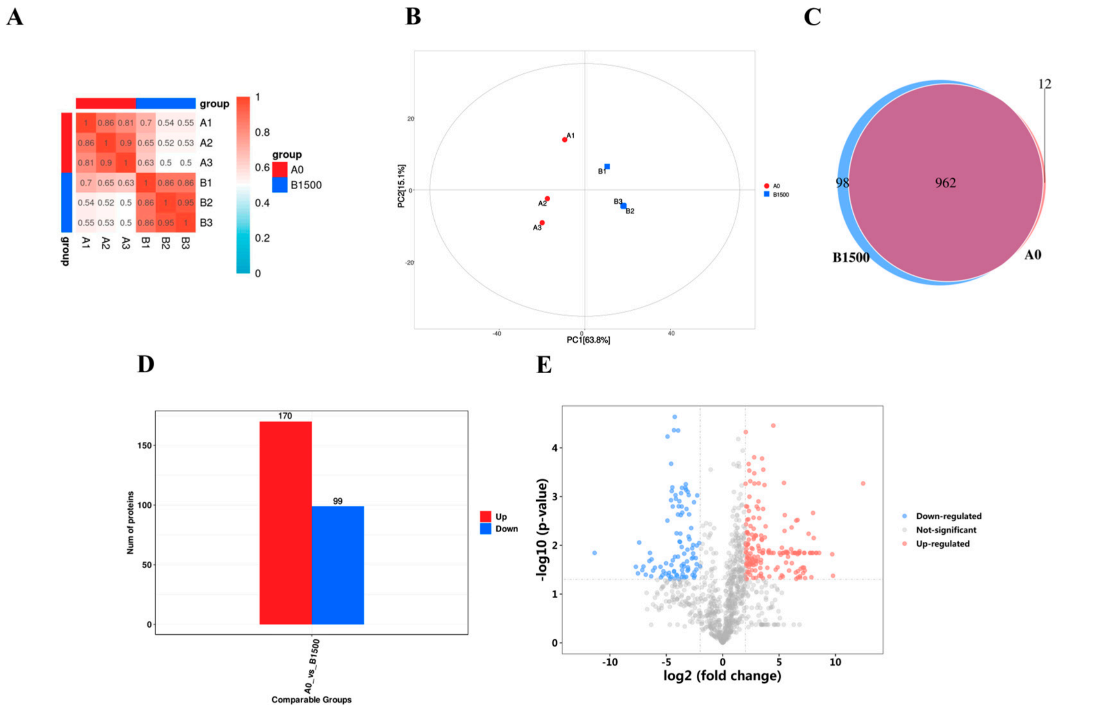

Figure S2. Basic Proteomic Sample Characteristics; (A) Heat map of inter-sample correlation; (B) Scatterplot of PCA scores for all samples; (C) Venn Diagram for Repeatability Analysis of Intergroup Sample Identification; (D) Histogram of the distribution of the number of differentially expressed proteins in different comparison groups; (E) Group A0 vs. Group B1500 Volcano Map.

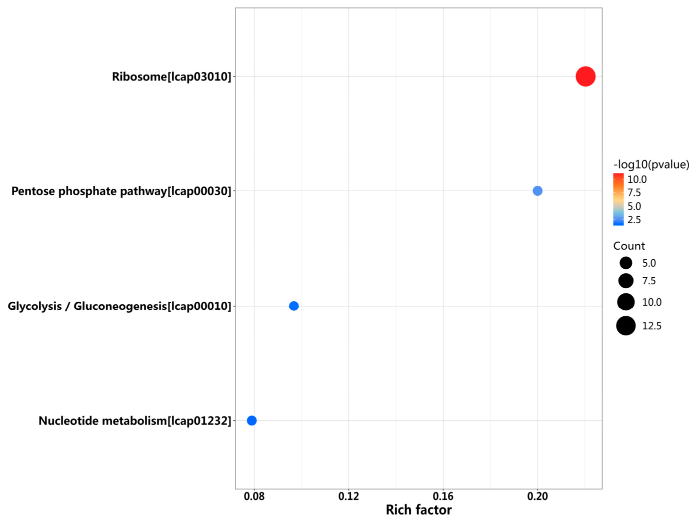

Figure S3. Enrichment analysis of KEGG metabolic pathway for differentially expressed proteins: bubble map
